# Metabolic fingerprinting for discrimination of DNA-authenticated *Atractylodes* plants using ^1^H NMR spectroscopy

**DOI:** 10.1007/s11418-020-01471-0

**Published:** 2021-02-11

**Authors:** Tatsuya Shirahata, Hiroshi Ishikawa, Teruhisa Kudo, Yumiko Takada, Azusa Hoshino, Yui Taga, Yusaku Minakuchi, Tomoko Hasegawa, Rina Horiguchi, Takehiro Hirayama, Takahiro Konishi, Hiroaki Takemoto, Noriko Sato, Masako Aragane, Tetsuro Oikawa, Hiroshi Odaguchi, Toshihiko Hanawa, Eiichi Kodaira, Tatsuo Fukuda, Yoshinori Kobayashi

**Affiliations:** 1 School of Pharmacy, Kitasato University, 5-9-1 Shirokane, Minato-ku, Tokyo, 108-8641 Japan; 2 Kitasato University Oriental Medicine Research Center, Kitasato University, 5-9-1 Shirokane, Minato-ku, Tokyo, 108-8641 Japan; 3 Tokyo Metropolitan Institute of Public Health, 24-1 Hyakunin-chou, 3-chome, Shinjuku-ku, Tokyo, 169-0073 Japan

**Keywords:** *Atractylodes* plants, ITS sequence, ^1^H NMR spectroscopy, Metabolic profiling

## Abstract

**Abstract:**

Identifying different species of the genus *Atractylodes* which are commonly used in Chinese and Japanese traditional medicine, using chromatographic approaches can be difficult. ^1^H NMR metabolic profiling of DNA-authenticated, archived rhizomes of the genus *Atractylodes* was performed for genetic and chemical evaluation. The ITS region of the nuclear rDNA was sequenced for five species, *A. japonica*, *A. macrocephala*, *A. lancea*, *A. chinensis*, and *A. koreana*. Our samples had nucleotide sequences as previously reported, except that part of the *A. lancea* cultivated in Japan had a type 5, hybrid DNA sequence. Principal component analysis (PCA) using ^1^H NMR spectra of extracts with two solvent systems (CD_3_OD, CDCl_3_) was performed. When CDCl_3_ extracts were utilized, the chemometric analysis enabled the identification and classification of *Atractylodes* species according to their composition of major sesquiterpene compounds. The ^1^H NMR spectra using CD_3_OD contained confounding sugar peaks. PCA removal of these peaks gave the same result as that obtained using CDCl_3_ and allowed species distinction. Such chemometric methods with multivariate analysis of NMR spectra will be useful for the discrimination of plant species, without specifying the index components and quantitative analysis on multi-components.

**Graphic abstract:**

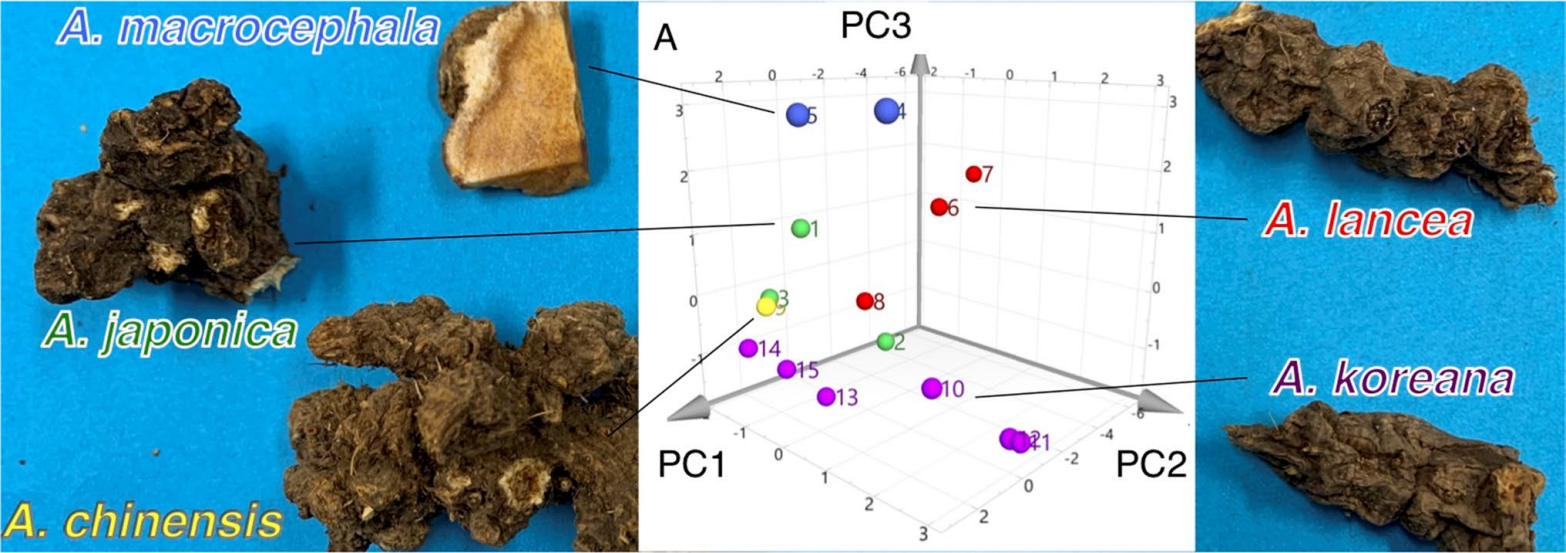

**Supplementary Information:**

The online version contains supplementary material available at 10.1007/s11418-020-01471-0.

## Introduction

Atractylodes rhizome (Atractylodis rhizoma, “Byakujutsu” in Japanese) and Atractylodes lancea rhizome (Atractylodis lanceae rhizoma, “Sojutsu” in Japanese) are often used as crude drugs in traditional Chinese medicine (TCM) and traditional Japanese medicine (Kampo). These plants, belonging to the genus *Atractylodes* and the family Compositae, are perennial herbs distributed in East Asia [1]. These two crude drugs have been used for the opposite clinical purposes in Kampo medicine; Byakujutsu for diaphoretic activity and Sojutsu has antisudorific activity [2, 3].

In the Japanese Pharmacopoeia, Atractylodes rhizome is defined as the dried rhizomes of *A. japonica* Koidzumi ex Kitamura and *A. macrocephala* Koidzumi (syn.: *A. ovata* De Candolle), whereas Atractylodes lancea rhizome is defined as the dried rhizomes of *A. lancea* De Candolle, *A. chinensis* Koidzumi, and the hybrid between those two species [4]. *A. lancea*, *A. chinensis*, *A. macrocephala*, and *A. koreana* grow wild in Northern China from the midstream reaches of the Yangtze River, northeast toward Inner Mongolia, while *A. japonica* is native to Japan. In the Korean Peninsula, *A. japonica* and *A. koreana* (Nakai) Kitamura grow wild [1]. The rhizome of *A. koreana* is sometimes used as a medicine in limited areas of China [5].

In the Japanese Pharmacopoeia, the two crude drugs are discriminated based on their morphological features [5] as well as by detection of the sesquiterpenoid atractylon [6], which is found only in Atractylodes rhizome (*A. macrocephala* and *A. japonica*). However, some varieties of *A. chinensis* have also been found to contain atractylon [7]. In addition, it is difficult to discriminate *A. lancea* and *A. chinensis* because of their overlap in morphological and chemical features [8, 9]. The differentiation of phenotypic trait between *Atractylodes* plants, is affected by environmental factors, such as the developmental stage [10], season of harvest [11], geographic origin of the plant [12], as well as by intra-specific variation [13–15], that makes the discrimination of *Atractylodes*-derived crude drugs difficult. By the use of genetic markers, four species of *Atractylodes* (*A. japonica*, *A. macrocephala*, *A. lancea*, and *A. chinensis*) were distinguished by their internal transcribed spacer (ITS) sequences. However, *A. koreana* could not be distinguished from *A. chinensis* by the ITS sequences alone [16].

Chromatographic fingerprinting (e.g., with HPLC or GC), can be used to clarify the required chemical characteristics of the species [17, 18]. However, the pre-purification step for chromatographic fingerprinting cannot detect all the metabolites present in the crude drug.

NMR spectroscopy can solve the common problems of chromatographic approaches. In recent years, NMR-based metabolomics technology has been successfully applied in many areas, including the analysis of herbal medicines, due to its unique advantages: (1) simple sample preparation, (2) high robustness, (3) simultaneous detection of primary and secondary metabolites, and (4) specific structural characterization [19–23]. The principal objective of this study was to establish a compound-based discrimination method for the rhizomes of five *Atractylodes* species, which are used in traditional Chinese and Japanese medicine. Thus, we performed ^1^H NMR metabolic profiling of these *Atractylodes* species, comparing the authentication with DNA barcoding. We could differentiate the five *Atractylodes* species by comparing the nucleotide sequences of the ITS region. ^1^H NMR metabolic profiling of the rhizome extracts with principal component analysis (PCA) also clearly discriminated these five *Atractylodes* species. We believe that our study will improve quality control of herbal medicine because our chemometric model (particularly the multivariate analysis using NMR measurements) will be useful for the assessment of these plant species and potentially others.

## Results and discussion

### Sequencing and phylogenetic analysis

The ITS sequences of specimens from the five *Atractylodes* species were determined and compared with previous data from the International Nucleotide Sequence Database (INSD) Table 1. Table 2 shows the nucleotide differences among various ITS sequences derived from *Atractylodes* plants. All sequences were of the same length, in which the ITS region, containing ITS1, the 5.8S rRNA gene, and ITS2, was 643 bp. The ITS sequences of No 1, 2, 3 plants were almost concordant with the Type 1 sequence (AB219405) in the previous study [12], which represents the *A. japonica* genotype; there were some differences among individuals, but they did not affect species definition. The ITS sequences of No 4, 5, No 6, 7, 8, and No 9 were concordant with the Type 2 (AB219406: *A. macrocephala*), Type 3 (AB219407: *A. lancea*), and Type 4 (AB219408: *A. chinensis* and AB219409: *A. koreana*) sequences, respectively. The ITS sequences of No 10, 11, 12 and No 13, 14, 15, morphologically identified as *A. koreana*, were almost identical to the Type 4 sequence and were indistinguishable from *A. chinensis* by the ITS genotype, as reported previously [12].

**Table 1:**
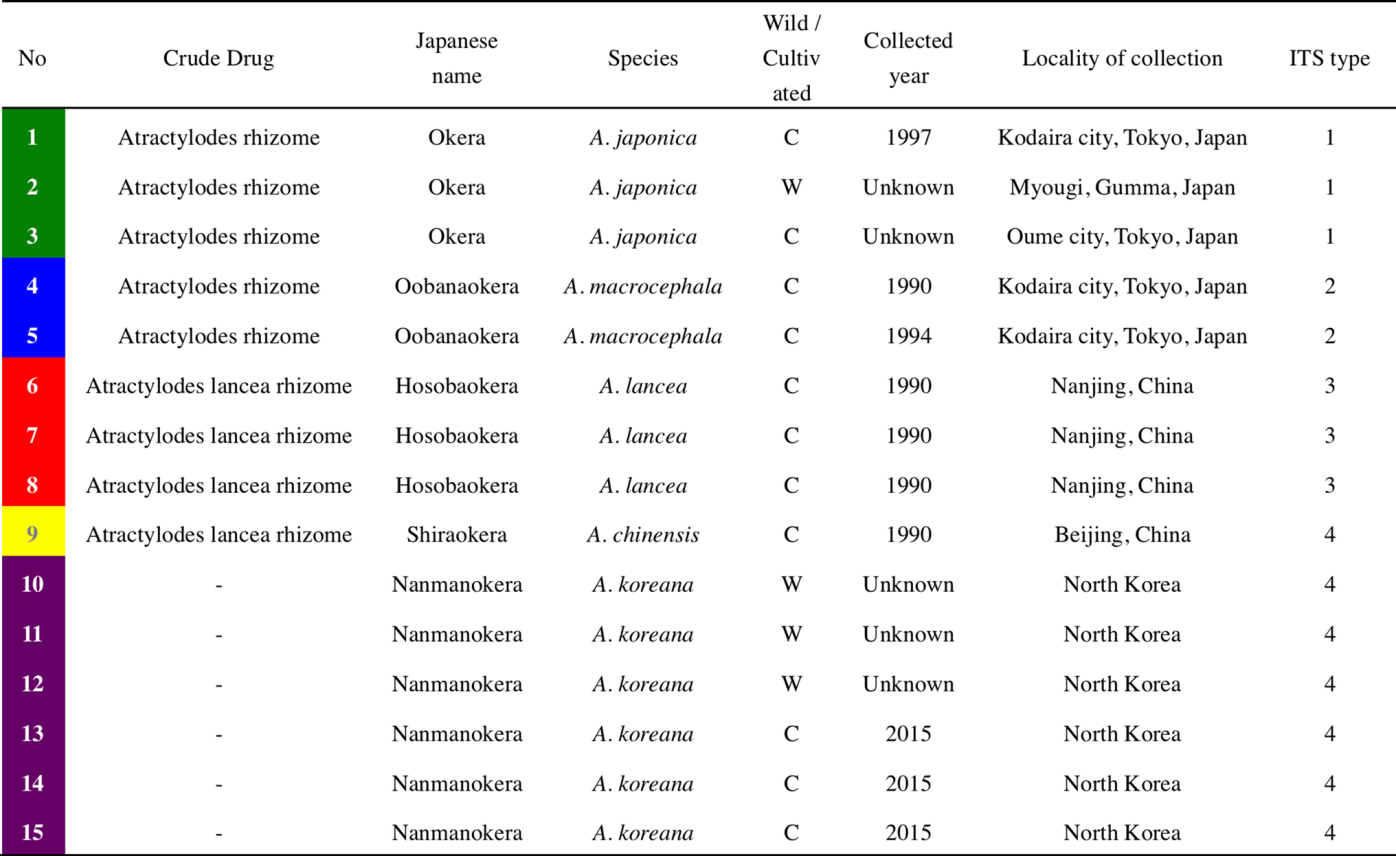
Plant specimens used in this study. The *Atractylodes* spp. used in this study were shown in different colors for each species: *A. japonica* in shown in green, *A. macrocephala* in blue, *A lancea* in red, *A. chinensis* in yellow, and *A. koreana* in violet. These colors are consistent with other figures and tables in this paper

**Table 2:**
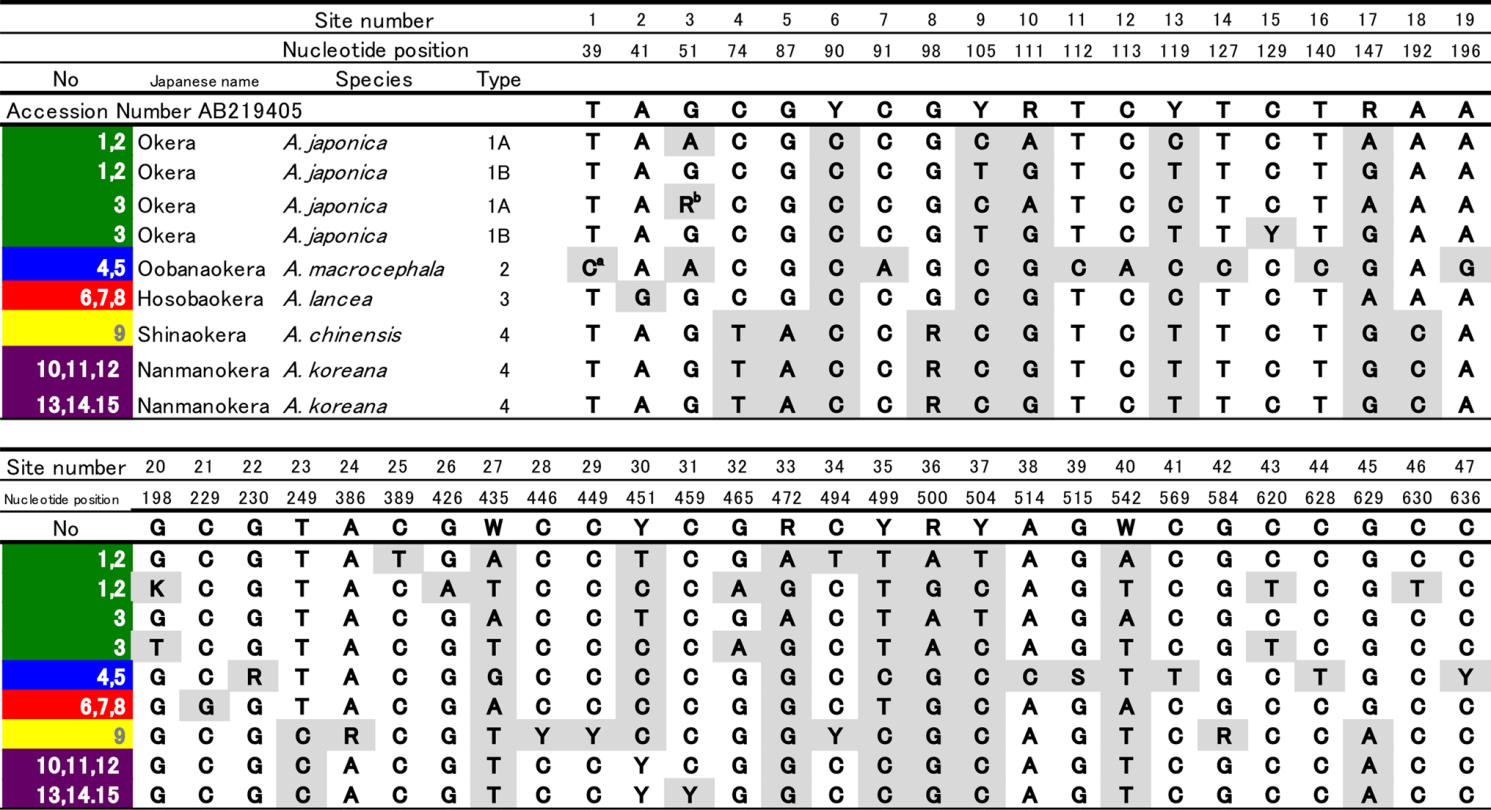
Comparison of ITS sequences among various samples derived from *Atractylodes* plants

^a^Gray background indicates nucleotide sites different from accession number AB219405

^b^Characters other than A, C, G, and T indicate nucleotide additives. R = A + G, W = A + T, S = C + G, Y = C + T, K = G + T

The ITS sequences of the isolated haplotypes from the No 1, 2, 3 were classified into two types, Type 1A and Type 1B, based on sequence similarity. Each individual possessed both types of ITS sequences. The Type 1 sequence can be interpreted as a combination of the two types. This suggests that *A. japonica* is of hybrid origin between unknown species with Type 1A and Type 1B sequences. Further investigations are needed to verify this hypothesis regarding the origin of *A. japonica*.

**Fig. 1:**
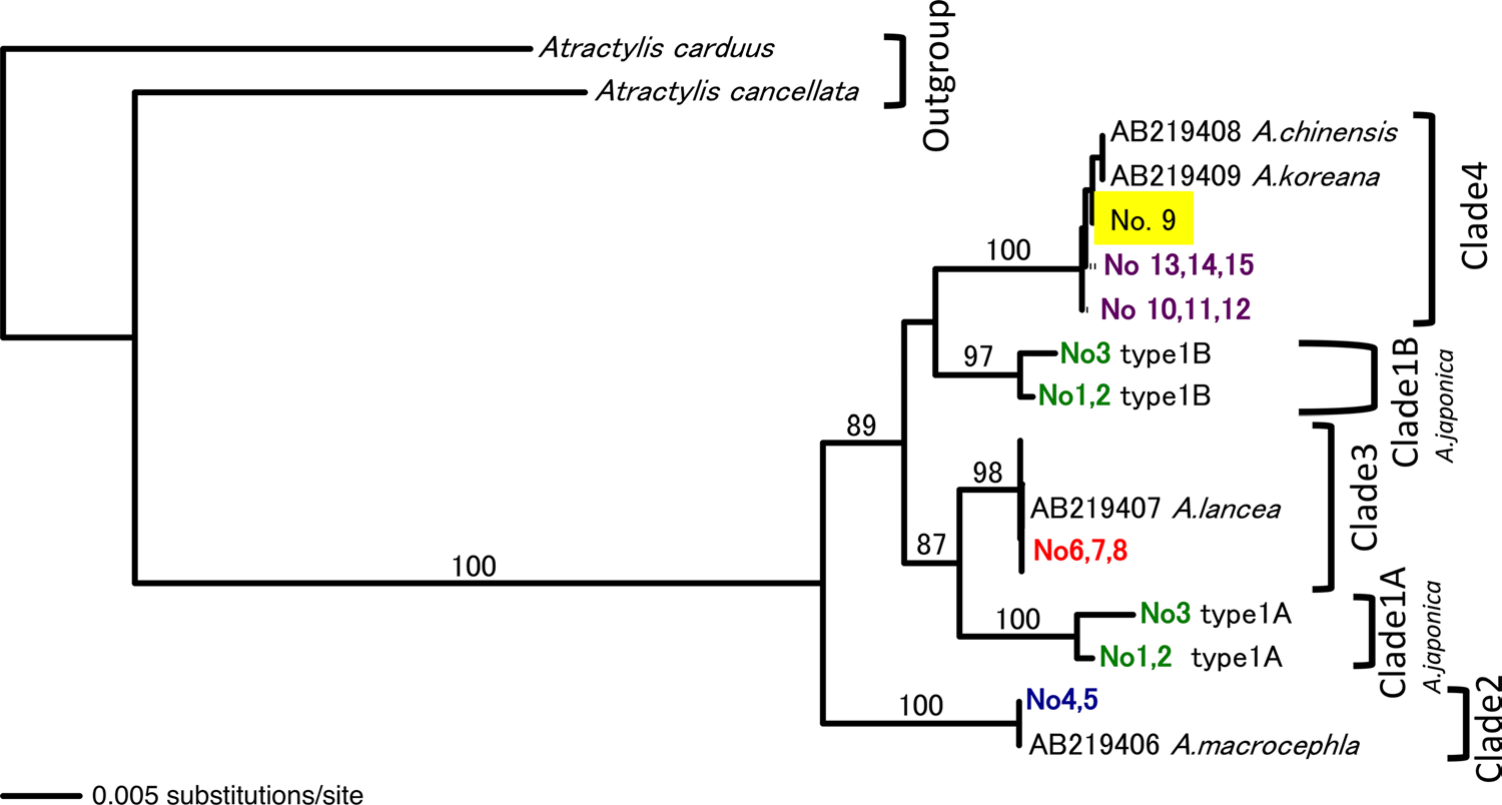
The NJ tree based on the ITS sequences of *Atractylodes* species. The bootstrap values above 80% are shown along the branches

Phylogenetic trees based on the ITS sequences of *Atractylodes* species were constructed with three different methods [neighbor joining (NJ), maximum parsimony (MP), and maximum likelihood (ML)]. Figure 1 shows the NJ tree of the ITS sequences. Five clades (clade 1A, 1B, 2, 3, and 4) were resolved in the ingroup. These five clades correspond to the Type 1A haplotype of *A. japonica*, Type 1B haplotype of *A. japonica*, Type 2 sequence: *A. macrocephala*, Type 3 sequence: *A. lancea*, and Type 4 sequence: *A. chinensis* and *A. koreana*, respectively. All five clades are well supported with more than 97% bootstrap values. The MP and the ML trees showed the same topology as the NJ tree except for differences in the relationships among operational taxonomic units in clade 4, which is beyond the scope of the current study. Thus, the ITS sequence was sufficiently informative to discriminate four *Atractylodes* species (*A. japonica*, *A. macrocephala*, *A. lancea*, and *A. chinensis*). However, *A. chinensis* and *A. koreana*, could not be distinguished solely by the ITS sequence.

### Identification of metabolites from *Atractylodes* plants

**Fig. 2:**
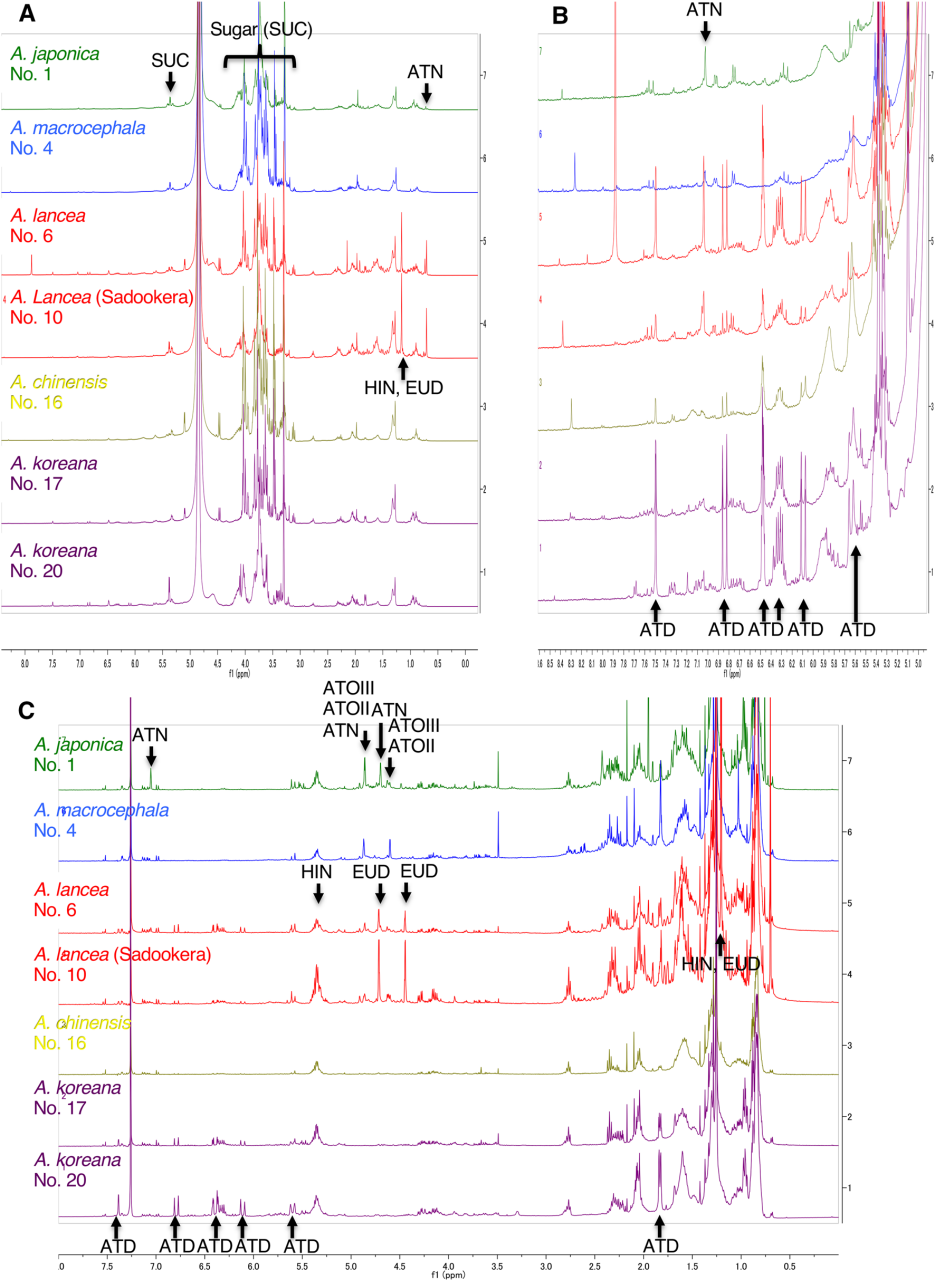
Representative ^1^H-NMR spectra of **a** CD_3_OD and **c** CDCl_3_ extracts of *Atractylodes* plants. **b** Expanded spectral region from 0.60 to 1.8 ppm of CD_3_OD extract. Signal numbers follow those listed in Table3 for metabolite identification using ^1^H NMR

**Fig. 3:**
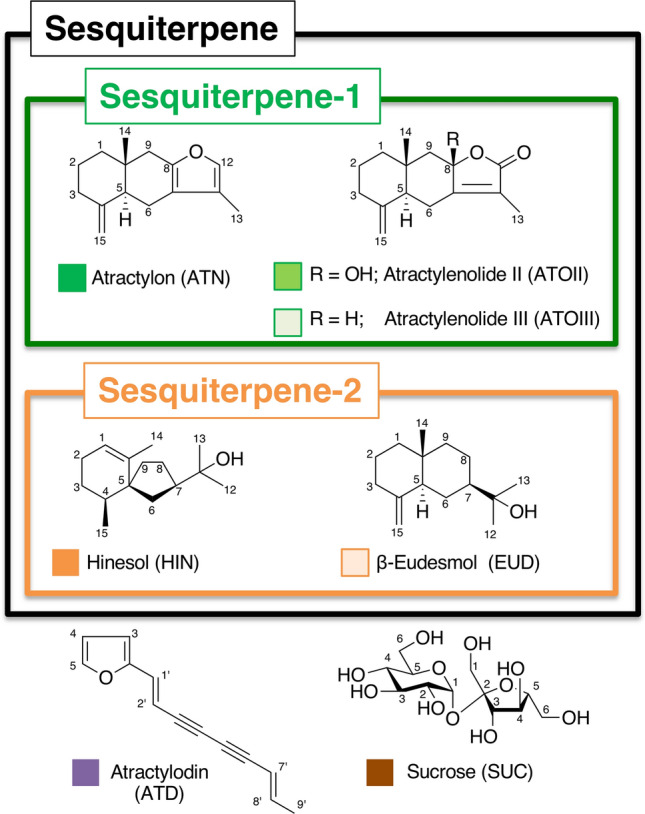
Chemical structures of the major components identified from *Atractylodes* plants

The ^1^H NMR spectra of the CD_3_OD extracts of the five species are shown in Fig. 2a. We assigned signals to seven compounds the chemical structures of which are shown in Fig. 3. The color cubic signs beside each chemical name relate to the corresponding buckets in the loading plot in PCA analysis (vide infra) in Fig. 3. Detailed peaks were assigned to the metabolites by comparisons to pure, isolated compounds as shown in Table 3, except sucrose in CDCl_3_. These assignments included five sesquiterpenoids, atractylon (ATN), atractylenolide II (ATOII), atractylenolide III (ATOIII), hinesol (HIN), and β-eudesmol (EUD), a polyacetylenic compound, atractylodin (ATD), and a sugar, sucrose (SUC). In the ^1^H NMR spectra using the CD_3_OD extracts, the main peaks were assigned to sucrose (δ 5.35, δ 4.00–3.30). In addition, H-12 and H-13 of hinesol and β-eudesmol were identified as a singlet at δ 1.16. Expanded spectra in the lower field were required to clarify each characteristic peak (Fig. 2b). The H-12 peak of atractylon characteristically appeared as a singlet at δ 7.40. A polyacetylated compound, atractylodin, was also detected in the olefinic region at δ 7.49 (d, *J* = 1.5 Hz), 6.84 (d, *J* = 16.0 Hz), 6.48 (d, *J* = 3.3 Hz), 6.47 (dd, *J* = 3.3, 1.5 Hz), 6.32 (dd, *J* = 15.5, 6.9 Hz), 6.10 (d, *J* = 16.0 Hz), and 5.64 (ddd, *J* = 15.5, 1.8, 1.0 Hz). In the CD_3_OD spectra, only the above peaks were recognized in the standard compounds. In contrast, many characteristic peaks of each compound were identified in the CDCl_3_ extract spectra (Fig. 2c). The H-15 peaks of the characteristic sesquiterpenoids in *A. japonica* and *A. macrocephala* were observed as doublets or doublets of doublets at δ 4.87–4.56, such as atractylon (δ 4.86, 4.70), atractylenolide II (δ 4.86, 4.56), and atractylenolide III (δ 4.87, 4.60). These types of buckets were called “sesquiterpenoid-1” and indicated by green squares. To identify *A. lancea* and *A. chinensis*, major sesquiterpenoids were observed, such as the H-1 of hinesol (δ 5.31), and H-15 of β-eudesmol (δ 4.72, 4.45), according to their characteristic signals (Table 3). These types of buckets were called “sesquiterpenoid-2” and shown by orange squares. The H-15 of atractylon (δ 7.05), H-12 and H-13 of hinesol and β-eudesmol (δ 1.21), and the olefinic proton of atractylodin (δ 7.49, 6.84, 6.48, 6.47, 6.32, 6.10, and 5.64) showed the same pattern as with the CD_3_OD extract.

**Table 3:** ^1^H-NMR chemical shifts (δ) and coupling constant (*J*, Hz) of *Atractylodes* plants

Number	Compound		CD_3_OD				CDCL_3_			
			Position	^1^HNMR			Position	^1^HNMR		
	Atractylon	ATN	12	7.04	s		12	7.05	D	*J *= 1.0 Hz
			15a	4.80	d	*J* = 1.6 Hz	15a	4.86	Dd	*J* = 3.5, 1.6 Hz
			15b	4.71	d	*J* = 1.6 Hz	15b	4.70	Dd	*J* = 3.2, 1.6 Hz
			3,6,9	2.42-2.02	m		3,6,9	2.44-2.00	M	
			13	1.92	s		13	1.95	D	*J* = 1.0 Hz
			1,2,5	1.70-1.49	m		1,2,5	1.72-1.45	M	
			14	0.74	s		14	0.76	S	
	Atractylenolide II	ATOII	8	4.97	dd	*J* = 11.5, 6.4 Hz	8	4.79	Dd	*J* = 11.7, 6.5 Hz
			15a	4.87	d	*J* = 1.4 Hz	15a	4.86	D	*J* = 1.4 Hz
			15b	4.65	d	*J* = 1.4 Hz	15b	4.56	D	*J* = 1.4 Hz
			6a	2.77	dd	*J* = 13.9, 3.9 Hz	6a	2.68	Dd	*J* = 14.0, 3.9 Hz
			6b	2.43	m		3a	2.36-2.29	M	
			3a	2.37	m		6b	2.28	M	
			9a	2.28	dd	*J* = 12.0, 6.4 Hz	9a	2.25	Dd	*J* = 12.4, 6.5 Hz
			3b	2.06-1.98	m		3b	1.98-1.89	M	
			5	1.90	m		5	1.82-1.79	M	
			13	1.78	t	*J* = 1.8 Hz	13	1.78	T	*J* = 1.6 Hz
			1a, 2	1.66-1.55	m		1a, 2	1.62-1.53	M	
			1b	1.40-1.33	m		1b	1.27	dt	*J* = 12.9, 5.0 Hz
			9b	1.10	dd	*J* = 12.0, 11.5 Hz	9b	1.10	dd	*J* = 12.4, 11.7 Hz
			14	0.92	s		14	0.86	s	
	Atractylenolide III	ATOIII	15a	4.85	d	*J* = 1.4 Hz	15a	4.87	d	*J* = 1.3 Hz
			15b	4.63	d	*J* = 1.4 Hz	15b	4.6	d	*J* = 1.3 Hz
			6a	2.66	dd	*J* = 13.1, 3.1 Hz	6a	2.66	dd	*J* = 13.2, 3.2 Hz
			6b	2.42	m	*J* = 13.1, 1.5 Hz	6b	2.45	dd	*J* = 13.2,1.5 Hz
			3a	2.38-2.33	m		3a	2.01-1.92	m	
			9a	2.21	d	*J* = 13.3 Hz	9a	2.26	d	*J* = 13.6 Hz
			3b	2.06-1.96	m		3b	2.01-1.92	m	
			5	1.91-1.87	m		5	1.86-1.82	m	
			13	1.78	d	*J* = 1.4 Hz	13	1.82	d	*J* = 1.3 Hz
			2	1.69-1.60	m		1b,2a,2b	1.72-1.55	m	
			1b	1.56-1.51	m		9b	1.56	d	*J* = 13.3 Hz
			9b	1.47	d	*J* = 13.3 Hz	1a	1.24	dt	*J* = 11.8, 6.4 Hz
			1a	1.29	dt	*J* = 13.1, 5.1 Hz	14	1.03	s	
			14	1.04	s					
	Hinesol	HIN	1	5.28	m		1	5.31	m	
			2	2.00-1.90	m		2	1.96-1.90	m	
			3,4,6,7,8,9	1.79-1.28	m		3,4,6,7,8,9	1.78-1.25	m	
			14	1.68	m		14	1.68	m	
			12	1.16	s		12	1.21	s	
			13	1.16	s		13	1.21	s	
			15	0.92	d	*J* = 7.0 Hz	15	0.92	d	*J* = 6.5 Hz
	b-Eudesmol	EUD	15a	4.69	dd	*J* = 3.2, 1.5 Hz	15a	4.72	dd	*J* = 3.2, 1.6 Hz
			15b	4.45	dd	*J* = 3.2, 1.5 Hz	15b	4.45	dd	*J* = 3.2, 1.6 Hz
			3a	2.30	ddt	*J* = 13.1, 3.7, 1.5 Hz	3a	2.31	ddt	*J* = 13.0, 3.8, 1.6 Hz
			3b	2.01	m		3b	2.00	m	
			1a	1.77	m		1a	1.77	m	
			1b,2,5,6,7,8,9	1.68-1.14	m		1b,2,5,6,7,8,9	1.66-1.18	m	
			12	1.16	s		12	1.21	s	
			13	1.16	s		13	1.21	s	
			14	0.71	s		14	0.70	s	
	Atractylodin	ATD	5	7.49	d	*J* = 1.5 Hz	5	7.38	d	*J* = 1.7 Hz
			1'	6.84	d	*J* = 16.0 Hz	1'	6.79	d	*J* = 15.5 Hz
			3	6.48	d	*J* = 3.3 Hz	4	6.42	dd	*J* = 3.5, 1.7 Hz
			4	6.47	dd	*J* = 3.3, 1.5 Hz	3	6.37	d	*J* = 3.5 Hz
			8'	6.32	dq	*J* = 15.5, 6.9 Hz	8'	6.33	dq	*J* = 15.8, 6.9 Hz
			2'	6.10	d	*J* = 16.0 Hz	2'	6.11	d	*J* = 15.5 Hz
			7'	5.64	ddd	*J* = 15.5, 1.8, 1.0 Hz	7'	5.6	ddd	*J* = 15.8, 1.8, 1.0 Hz
			9'	1.82	dd	J = 6.9, 1.8 Hz	9'	1.83	dd	J = 6.9, 1.8 Hz
	sucrose	SUC	H-1	5.38	d	J= 3.8 Hz				
			H-3’	4.09	d	J= 8.1 Hz				
			H-4’	4.01	dd	J= 8.1, 7.9 Hz				
			H-3,5,6,5’,6’	3.84-3.67	m					
			H-1'	3.63	d	J= 12.1 Hz				
			H-1'	3.59	d	J= 12.1 Hz				
			H-2	3.41	dd	J= 9.8, 3.8 Hz				
			H-4	3.37-3.34	m					

### Principal component analysis using ^1^H NMR spectra of CD_3_OD extracts (CD_3_OD model)

**Fig. 4:**
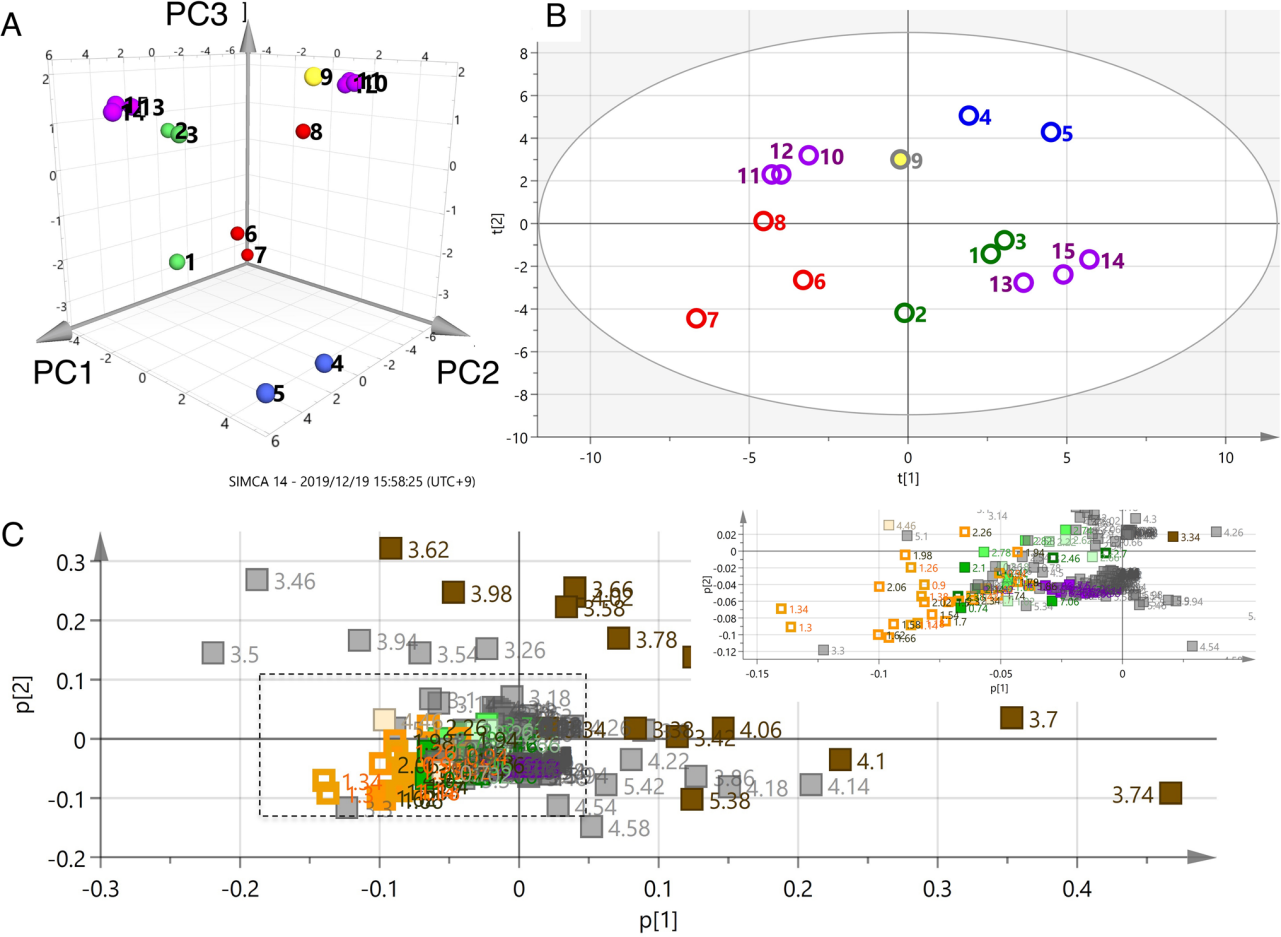
Principal component analyses (PCA) using ^1^H-NMR spectrum extracted with CD_3_OD of different *Atractylodes* samples. **a** 3D score plot of PC1, PC2 and PC3 scores, **b** score plot of PC1 and PC2 scores, **c** loading plot for PC1 and PC2 components

Principal component analysis is a widely applied unsupervised method in multivariate data analysis that aims to reduce the dimensionality of a multivariate dataset [24]. In this study, PCA was used for species classification and identification of marker metabolites. The bucketed data derived from the ^1^H NMR spectra of these samples were subjected to PCA. The data were scaled using the Pareto scaling method, which lies in-between non-scaling and the unit variance method [24, 25]. PCA extracted three significant principal components cumulatively accounting for 83% of the total variance (PC1 = 45%, PC2 = 26%, PC3 = 12%), which is shown in a 3D score plot (Fig. 4a), where each point represents an individual sample. A partial separation was observed between *A. lancea* (red) and the samples from the other four species. To understand the differentiation of *A. lancea* in more detail, the score plot of the first two principal components generated from the 15 samples is shown in Fig. 4b. Two samples of *A. macrocephala* (blue) were adjacently located. The *A. koreana* samples (purple) were not clustered as one group, but other groups were clustered based on their species. The loading plot was analyzed to clarify the buckets that contributed to form these groupings (Fig. 4c). The buckets of sucrose (3–5 ppm), including the anomeric proton at 5.18 ppm, were spread on the far right side (PC1 positive value). The buckets defined as the sesquiterpene-2 group were found in the third quadrant, where the characteristic bucket from the protons of the dimethyl group at δ 1.16 ppm (s) was also found. Secondary metabolite components from the essential oil of Atractylodes lancea rhizome contributed to the grouping of samples from *A. lancea*. For the further consideration of this model, the score and lording plot of PC1 and PC3 were studied, which showed that sesquiterpene-1 and -2 were not clustered on the lording plot. The PC1 and PC3 data were showed in the Electronic supplementary material (ESM)(Figures S1, S2 and S3, vide infra). Unfortunately, these PCA models could not differentiate the plant species due to the confounding resonances of sucrose (3–5 ppm). As a second step, PCA was performed using the ^1^H NMR spectra of compounds extracted with a low polar solvent to prevent detection of sucrose and allow the delineation of the plant species.

### Principal component analysis using ^1^H NMR spectra of CDCl_3_ extracts (CDCl_3_ model)

**Fig. 5:**
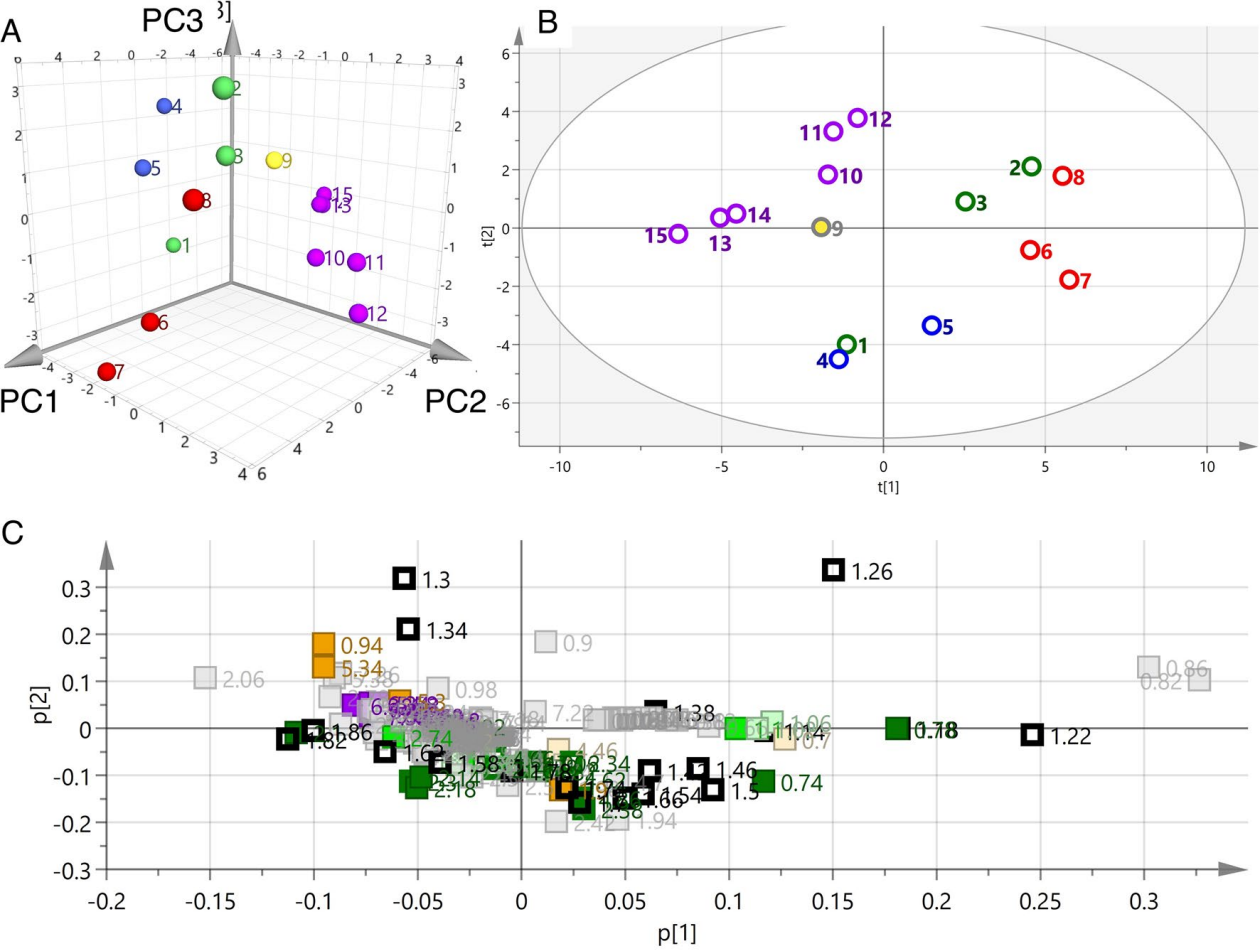
Principal component analyses (PCA) using ^1^H-NMR spectrum extracted with CDCl_3_ of different *Atractylodes* samples. **a** 3D score plot of PC1, PC2 and PC3 scores **b** score plot of PC1 and PC2 scores **c** loading plot for PC1 and PC2 components

**Fig. 6:**
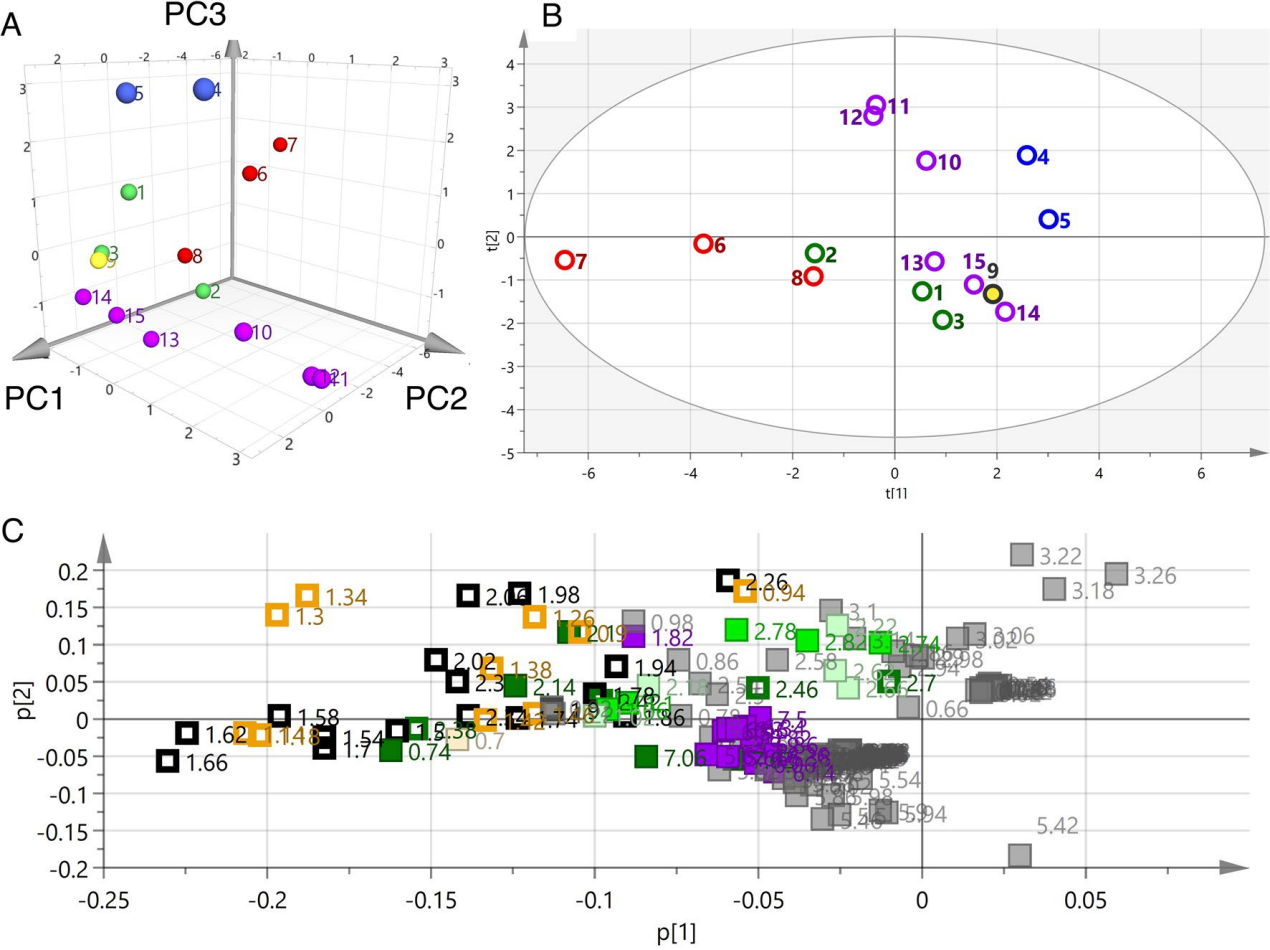
Principal component analyses (PCA) using ^1^H-NMR spectrum removed a sugar-based region (δ 3.30–5.40) extracted with CD_3_OD of different *Atractylodes* samples. **a** 3D score plot of PC1, PC2 and PC3 scores, **b** score plot of PC1 and PC2 scores, **c** loading plot for PC1 and PC2 components

PCA using ^1^H NMR spectra of *Atractylodes* samples extracted with CDCl_3_, which is unaffected by primary metabolites, was performed utilizing bucket integral values every 0.04 ppm in the range of 0.00–10.00 ppm. The results showed three significant principal components cumulatively accounting for 82% of the total variance (PC1 = 50%, PC2 = 21%, PC3 = 11%), which are presented in the 3D score plot in Fig. 6a. The 3D score plot in Fig. 5b, shows three major, distinct clusters corresponding to the different species studied. The samples of *A. koreana* (purple) were clustered in one group, unlike the CD_3_OD extracts. The score plot of PC1/PC2 was analyzed to understand the details of the clustering (Fig. 5b). Plots of *A. koreana* were found in the second quadrant. The PC1 and PC2 loading plots were studied further to clarify potential metabolic markers contributing to the discrimination of the different species (Fig. 5c). In the fourth quadrant, buckets of 0.74 and 0.78 were confirmed corresponding to the proton at C14 (0.76 ppm, s) in atractylon, which is a specific compound found in *Atractylodes* rhizome. In contrast, in the third quadrant, there are buckets derived from protons at 6.11, 6.33, 6.37, 6.42, 6.79, and 7.38 ppm, which are considered to be inherent to atractylodin from *A. koreana* (shown in purple). In the fourth quadrant, the buckets of the sesquiterpene-2 group derived from *Atractylodes lancea* rhizome were involved. In addition, the protons of the dimethyl group were found to be 1.22 ppm (s) deviated from the center. From the above, it was possible to distinguish the species on the basis of PCA plots by detecting known, base-like compounds by NMR and performing multivariate analysis (PCA).

### Principal component analysis excluding sugar-based buckets in ^1^H NMR spectra of CD_3_OD extracts (CD_3_OD sugar KO model)

Using deuterated chloroform made it possible to distinguish basic species without the influence of highly water-soluble primary metabolites, especially sugars (sucrose in this instance). Therefore, PCA was performed excluding the sugar region (3.30 to 5.40 ppm) of the ^1^H NMR spectra of the CD_3_OD extracts, because the PCA derived from the reduced NMR dataset was in general agreement with the PCA using CDCl_3_ extracts. The results showed three significant principal components cumulatively accounting for 83% of the total variance. The 3D score plot showed major distinct clusters corresponding to the five different species (Fig. 6a, the contributions of PC1, PC2, and PC3 were 46%, 19%, and 18%, respectively). Especially, samples of *A. koreana* (purple) formed a cluster similar to that from the CDCl_3_ extracts. The PC1/2 loading plot was used to verify group formation on the score plot (Fig. 6b). There were many sesquiterpene-2-group buckets such as the chemical shifts of the dimethyl group resonances in hinesol and β-eudesmol, appearing at 1.16 ppm in the fourth quadrant, and whose clustering supported the grouping of *A. lancea* on the score plot. In contrast, the sesquiterpene-1 group (green) bucket was positioned in the second quadrant in the loading plot, which was in agreement with the distribution of the *A. japonica* group on the score plot. The *A. koreana* samples were distinguished as one group by the presence of the bucket corresponding to the resonance of atractylodin.

Thus, by modifying the calculation method, it was possible to fully utilize the performance of ^1^H NMR metabolic profiling without changing the extraction solvent. In this method, highly water-soluble primary metabolites, such as saccharides, are excluded from the calculation to obtain characteristic metabolic fingerprints from the *Atractylodes* plants. On the other hand, this knocking out method would also exclude signals corresponding to glycosides at the same time. However, in this time, the KO model showed good agreement with the CDCl_3_ model for the discrimination of *Atractylodes* species. The reason for the discrimination is supposed to be that sucrose was the main component during the knockout region.

### Comparison between three models using calculation parameters

The merit of NMR metabolic profiling is that the classification of plant species can be performed without specifying the index components. We successfully established a suitable method for ^1^H NMR metabolic profiling of *Atractylodes* plants by selecting a bucket according to the desired parameters and performing statistical calculations with the same solvent.

The multiple correlation coefficient (*R*^2^) and predictive ability parameter (*Q*^2^) in each of the three PCA models, extraction of CD_3_OD, CDCl_3_ and CD_3_OD knockout of sugar region (CD_3_OD sugar KO), are shown in Table 4. Two-way orthogonal partial least squares (O2PLS-DA) was performed on the three models to add various classes of *Atractylodes* species as objective variables, checked the multiple correlation coefficient (*R*^2^) and predictive ability parameter (*Q*^2^) of the resulting model, and performed a permutation test. *R*^2^ and *Q*^2^ values indicate the model's linearity and predictive ability, respectively. A discrimination model with an *R*^2^ value of > 0.65 and a *Q*^2^ value of > 0.5 is regard as adequate for discrimination [31].

**Table 4:** Estimation index results for all the discrimination models

Model			Permutation test									
			*A. japonica*		*A. macrocephala*		*A. lancea*		*A. chinensis*		*A. koreana*	
	*R*^2^	*Q*^2^	*R*^2^	*Q*^2^	*R*^2^	*Q*^2^	*R*^2^	*Q*^2^	*R*^2^	*Q*^2^	*R*^2^	*Q*^2^
CD_3_OD	0.850	0.390	0.518	− 1.21	0.423	− 1.30	0.353	− 1.37	0.579	− 0.617	0.514	− 1.29
CDCL_3_	0.886	0.310	0.445	− 1.06	0.477	− 1.40	0.441	− 1.52	0.535	− 0.911	0.430	− 1.20
CD_3_OD sugar KO	0.871	0.480	0.386	− 1.21	0.398	− 0.858	0.402	− 1.02	0.495	− 0.521	0.423	− 1.02

A permutation test is used to validate the incidence of overfitting in a predictive model [32, 33]. In this test, provisional discrimination models were constructed on the basis of various data matrices in which objective and explanatory variables were randomly combined many times, and *R*^2^ and *Q*^2^ were calculated for each provisional model. The original and permuted data matrices and the correlation coefficients of *R*^2^ and *Q*^2^ were plotted on the *x* and *y* axes, respectively. The *y* intercept of the regression line in the plot is used as the estimated index for overfitting: generally, *R*^2^ < 0.4 and *Q*^2^ < 0.05 [32, 33]. Table 4 shows the results of each estimated index for all the discrimination models.

The value of R^2^Y did not change in any of the models, but the value of Q^2^Y showed a good fit in the CD_3_OD sugar KO model. This suggested that the CD_3_OD sugar KO model could be used as the discrimination model. From the results of the permutation test, it was found that using the CD_3_OD sugar KO model was suitable for predicting *A. japonica* and *A. macrocephala*.

## Conclusion

In conclusion, we performed ^1^H NMR metabolic profiling of DNA-authenticated, archived rhizomes of the *Atractylodes* genus for genetic and chemical quality evaluation. The nucleotide sequence of the ITS region of the nuclear rDNA was confirmed for five species, *A. japonica*, *A. macrocephala*, *A. lancea*, *A. chinensis*, and *A. koreana*. An unbiased approach using multivariate statistical analysis of ^1^H NMR spectra of CD_3_OD extracts was adopted to reveal compositional differences in the primary and secondary metabolites among *Atractylodes* species, however, we failed to discriminate these plant species by this condition. Therefore, we prepared analytical samples by CDCl_3_ extraction, in which clustering of each plant species was achieved to detect species-specific compounds on the score plot of the PCA. Removing the sugar peaks from the ^1^H NMR spectra of the CD_3_OD extracts with PCA gave the same results as the PCA using CDCl_3_ extracts. This biased chemometric model was able to successfully discriminate these plant species. The present study revealed that ^1^H NMR-based metabolic profiling and genetic assessment are useful to identify members of the *Atractylodes* genus, which are categorized as different drugs in the Japanese Pharmacopoeia.

## Experimental section

### General experimental procedures

Polymerase chain reactions (PCRs) were performed with Ex Taq DNA polymerase (Takara, Kyoto, Japan). Sequencing reactions were carried out with Big Dye Terminator v3.1 Cycle Sequencing Kits (Applied Biosystems, CA, USA), and the amplicons were electrophoresed on an ABI 3130 Genetic Analyzer (Applied Biosystems, CA, USA). All ground *Atractylodes* samples were extracted with an Accelerated Solvent Extraction system (ASE 350) from Dionex Corporation (Sunnyvale, CA, USA). The extracts were dried using a Thermo-Fisher Savant SC250EXP SpeedVac™ equipped with an RVT4104 refrigerated vapor trap. Freeze-drying was performed on a Labconco Freezone 4.5 (Kansas City, MO, USA). A precise Mettler Toledo XS105 dual range analytical balance was employed to prepare extracts for UHPLC and quantitative ^1^H NMR (qHNMR) analyses. Samples for NMR analyses were prepared with a Pressure-Lock gas syringe (VICI Precision Sampling Inc., Baton Rouge, LA, USA) and calibrated glass pipets (cat. no: 2-000-200, Drummond Scientific, Broomall, PA, USA). Standard NMR tubes of 3 mm × 7 in. were purchased from Shigemi Co., Ltd. (no. PS005, Hachioji-city, Tokyo, Japan). ^1^H NMR spectra were recorded on an Agilent Technologies 400-MR (400 MHz).

### Reagents

*Atractylodes* standards, β-eudesmol, and atractylenolide III were obtained from Fujifilm Wako Pure Chemical Co. (Osaka, Japan). For NMR acquisition, CD_3_OD-*d*_*4*_ (99.8% D) and CDCl_3_ (99.8% D) were purchased from Merck KGaA (Darmstadt, Germany). The signals in the ^1^H NMR spectra of *Atractylodes* extracts were assigned to individual metabolites on the basis of thorough analyses of the 2D NMR spectra and spiking experiments. The PCR-grade tubes, tips, and most biological reagents used for DNA authentication were acquired from Qiagen (Valencia, CA, USA) and/or Thermo-Fisher Scientific and Beckman Coulter (Indianapolis, IN, USA). LO3 agarose for gel electrophoresis was purchased from Takara (Kyoto, Japan).

### Plant material

Fifteen specimens of five *Atractylodes* species were analyzed: *A. japonica*, *A. macrocephala*, *A. lancea*, *A. chinensis*, and *A. koreana*. Details of the plant materials are shown in Table 1. Samples were botanically/macroscopically verified prior to inclusion. All the voucher specimens were deposited in Medicinal Plant Garden, School of Pharmacy, Kitasato University, Kanagawa, Japan.

### DNA extraction, PCR amplification, and sequencing

Total DNA was extracted from 100–200 mg of leaf tissue using hexadecyltrimethylammonium bromide (CTAB) solution following the method of Doyle [26] with minor modifications. The primer pair ITS5 (5′-GGA AGT AAA AGT CGT AAC AAG G-3′) and ITS4 (5′-TCC TCC GCT TAT TGA TAT GC-3′) [27] was used to amplify the ITS region of nrDNA. PCR amplification was performed in a 50-μL reaction volume containing 1 × reaction buffer for Ex Taq DNA polymerase, 0.2 mM of each dNTP, 0.2 μM of each primer, 0.5 units of Ex Taq DNA polymerase (Takara, Kyoto, Japan), and approximately 10 ng of template DNA. PCR was performed under the following cycling conditions: (95 °C, 3 min) × 1 cycle, (95 °C, 1 min; 52 °C, 1 min; 72 °C, 1 min 30 s) × 30 cycles, and (72 °C, 8 min) × 1 cycle. Since it is known that there are additive nucleotides in the ITS sequence of *A. japonica* [12], single strand conformation polymorphism (SSCP) analysis with the first PCR products was carried out to isolate individual alleles, following the method of Watano et al*. *[28]*.* Segregated bands were excised and purified before use as template DNA for the second PCR. The PCR products were purified with Amicon® Ultra Centrifugal Filter Units (Merck, Germany). Sequencing reactions using purified PCR products were carried out with BigDye Terminator v3.1 Cycle Sequencing Kits (Applied Biosystems, CA, USA) under the following cycling conditions: (96 °C, 1 min) × 1 cycle, (96 °C, 10 s; 50 °C, 5 s; 60 °C, 3 min 30 s) × 40 cycles, and (60 °C, 5 min) × 1 cycle. A specific sequence primer, AJITSF3 (5′-CCG CGA ACA TGT AAT GAC AAC CGG GC-3′) or AJITSR3 (5′-AAG CGT CGT CGC GAG GCG AC-3′) was used to avoid non-specific annealing. The reaction products were analyzed using the ABI3130 Genetic Analyzer (Applied Biosystems, CA, USA).

### Phylogenetic analysis

Sequence data were edited and aligned using BioEdit [29]. Phylogenetic analysis by three different methods (NJ, MP, and ML) was performed using PAUP 4.0b10 [30]. Bootstrap analysis of 1000 replicates was conducted for the NJ and MP trees.

### Sample extraction and preparation

Each *Atractylodes* plant sample was extracted using 0.1 mg of material in 1.0 mL of CD_3_OD-*d*_4_ (99.8% D) or CDCl_3_ (99.8% D) by ultrasonication at room temperature for 1 h. The mixture was centrifuged at 3,000 rpm (Kubota 3740, Japan) for 5 min. The supernatants were filtered through an Ekicrodisc® 30 mm syringe membrane filter (0.45-μm pore size) and transferred into 3-mm standard NMR tubes.

### ^1^H NMR acquisition and ^1^H NMR multivariate data analysis

The 1D ^1^H NMR spectra were acquired at 298 K using a 45° excitation pulse experiment (Bruker pulprog: zg). The probe was frequency-tuned and impedance-matched before each acquisition. For each sample, 64 scans (ns) and 4 dummy scans (ds) were recorded with the following parameters: spectral width of 16 ppm, relaxation delay (D1) of 3.0 s, and receiver gain (RG) set to 256. The total duration of each ^1^H NMR acquisition was 15 min. Off-line data processing was performed using MNova Lite (Mestrelab Research, S.L.) and ALICE2 software (JEOL). The ^1^H NMR spectra were automatically Fourier-transformed using ALICE2 software (JEOL). The spectra were referenced to CH_3_OH at 3.30 ppm and CHCl_3_ at 7.26 ppm. Spectral intensities were reduced to integrated regions, referred to as buckets, of equal width (0.04 ppm) within the region of δ 10.0 to 0.00 ppm using ALICE2 for metabolome version 6 software (JEOL). In the case of CD_3_OD, the region between δ 5.0 and 4.6, corresponding to residual water signals, was removed. The total integral value on the spectrum was set to 100 to provide bucket tables. In the resulting bucket tables, all rows were scaled to the total intensity, and Pareto scaling was applied to the columns preceding PCA and O2PLS-DA using SIMCA software. For another experiment, the regions between 4.00 and 3.00 ppm, corresponding to the sugar region, and the sucrose anomeric resonance at 5.169 ppm (doublet *J* = 3.75 Hz) were removed from the bucket table.

## Supplementary Information

Below is the link to the electronic supplementary material.

Supplementary file1 (PDF 3605 KB)
