# Correction to: Metabolic fingerprinting for discrimination of DNA-authenticated *Atractylodes* plants using ^1^H NMR spectroscopy

**DOI:** 10.1007/s11418-022-01616-3

**Published:** 2022-03-16

**Authors:** Tatsuya Shirahata, Hiroshi Ishikawa, Teruhisa Kudo, Yumiko Takada, Azusa Hoshino, Yui Taga, Yusaku Minakuchi, Tomoko Hasegawa, Rina Horiguchi, Takehiro Hirayama, Takahiro Konishi, Hiroaki Takemoto, Noriko Sato, Masako Aragane, Tetsuro Oikawa, Hiroshi Odaguchi, Toshihiko Hanawa, Eiichi Kodaira, Tatsuo Fukuda, Yoshinori Kobayashi

**Affiliations:** 1 School of Pharmacy, Kitasato University, 5-9-1 Shirokane, Minato-ku, Tokyo, 108-8641 Japan; 2 Kitasato University Oriental Medicine Research Center, Kitasato University, 5-9-1 Shirokane, Minato-ku, Tokyo, 108-8641 Japan; 3 Tokyo Metropolitan Institute of Public Health, 24-1 Hyakunin-chou, 3-chome, Shinjuku-ku, Tokyo, 169-0073 Japan

## Correction to: J Nat Med (2021) 75:475–488 10.1007/s11418-020-01471-0

The article “Metabolic fingerprinting for discrimination of DNA-authenticated *Atractylodes* plants using ^1^H NMR spectroscopy”, written by Tatsuya Shirahata, Hiroshi Ishikawa, Teruhisa Kudo, Yumiko Takada, Azusa Hoshino, Yui Taga, Yusaku Minakuchi, Tomoko Hasegawa, Rina Horiguchi, Takehiro Hirayama, Takahiro Konishi, Hiroaki Takemoto, Noriko Sato, Masako Aragane, Tetsuro Oikawa, Hiroshi Odaguchi, Toshihiko Hanawa, Eiichi Kodaira, Tatsuo Fukuda, Yoshinori Kobayashi, was originally published Online First without Open Access. After publication in volume 75, issue 3, pages 475–488 the author decided to opt for Open Choice and to make the article an Open Access publication. Therefore, the copyright of the article has been changed to © The Author(s) 2022 and the article is forthwith distributed under a Creative Commons Attribution 4.0 International License, which permits use, sharing, adaptation, distribution and reproduction in any medium or format, as long as you give appropriate credit to the original author(s) and the source, provide a link to the Creative Commons licence, and indicate if changes were made. The images or other third party material in this article are included in the article’s Creative Commons licence, unless indicated otherwise in a credit line to the material. If material is not included in the article’s Creative Commons licence and your intended use is not permitted by statutory regulation or exceeds the permitted use, you will need to obtain permission directly from the copyright holder. To view a copy of this licence, visit http://creativecommons.org/licenses/by/4.0/.

The original article has been updated.

