# Supplementary material for: Metabolic fingerprinting for discrimination of DNA-authenticated Atractylodes plants using 1H NMR spectroscopy
Source: J Nat Med. 2021 Feb 11;75(3):475–88. doi: 10.1007/s11418-020-01471-0 (PMC8924082; doi:10.1007/s11418-020-01471-0)
Supplement: Supplementary file 1 — Supplementary file1 (PDF 3605 KB) [file 11418_2020_1471_MOESM1_ESM.pdf]

Electronic supplementary material

Metabolic fingerprinting for discrimination of DNA-authenticated *Atractylodes* plants using ^{1}H NMR spectroscopy

Tatsuya Shirahata[†,‡], Hiroshi Ishikawa[†], Teruhisa Kudo[†], Yumiko Takada[†], Azusa Hoshino[†], Yui Taga[†], Yusaku Minakuchi[†], Tomoko Hasegawa[†], Rina Horiguchi[†], Takehiro Hirayama[†], Takahiro Konishi[†], Hiroaki Takemoto[†], Noriko Sato[†], Masako Aragane[§], Tetsuro Oikawa[‡], Hiroshi Odaguchi[‡], Toshihiko Hanawa[‡], Eiichi Kodaira[†], Tatsuo Fukuda[†], Yoshinori Kobayashi[†,‡]*

†School of Pharmacy, Kitasato University, 5-9-1 Shirokane, Minato-ku, Tokyo 108-8641, Japan.
‡ Kitasato University Oriental Medicine Research Center, Kitasato University, 5-9-1 Shirokane, Minato-ku, Tokyo 108-8641, Japan.
§ Tokyo Metropolitan Institute of Public Health, 24-1 Hyakuninn-chou, 3-chome, Shinjuku-ku, Tokyo 169-0073, Japan.

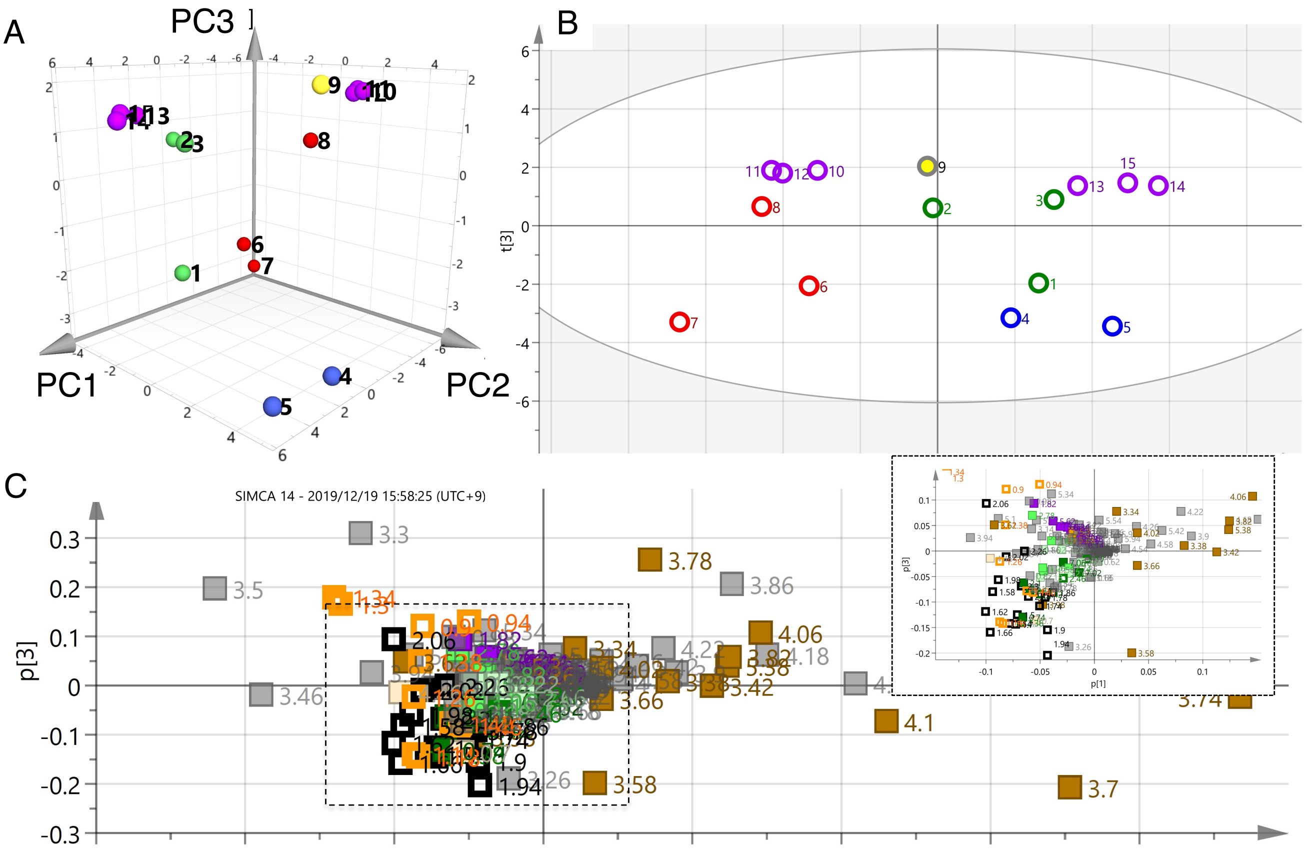

Figure S1. Principal component analyses (PCA) using ^{1}H-NMR spectrum extracted with CD_3OD of different *Atratylodes* samples. (A) 3D Score plot of PC1, PC2 and PC3 scores, (B) Score plot of PC1 and PC3 scores, (C) Loading plot for PC1 and PC3 components.

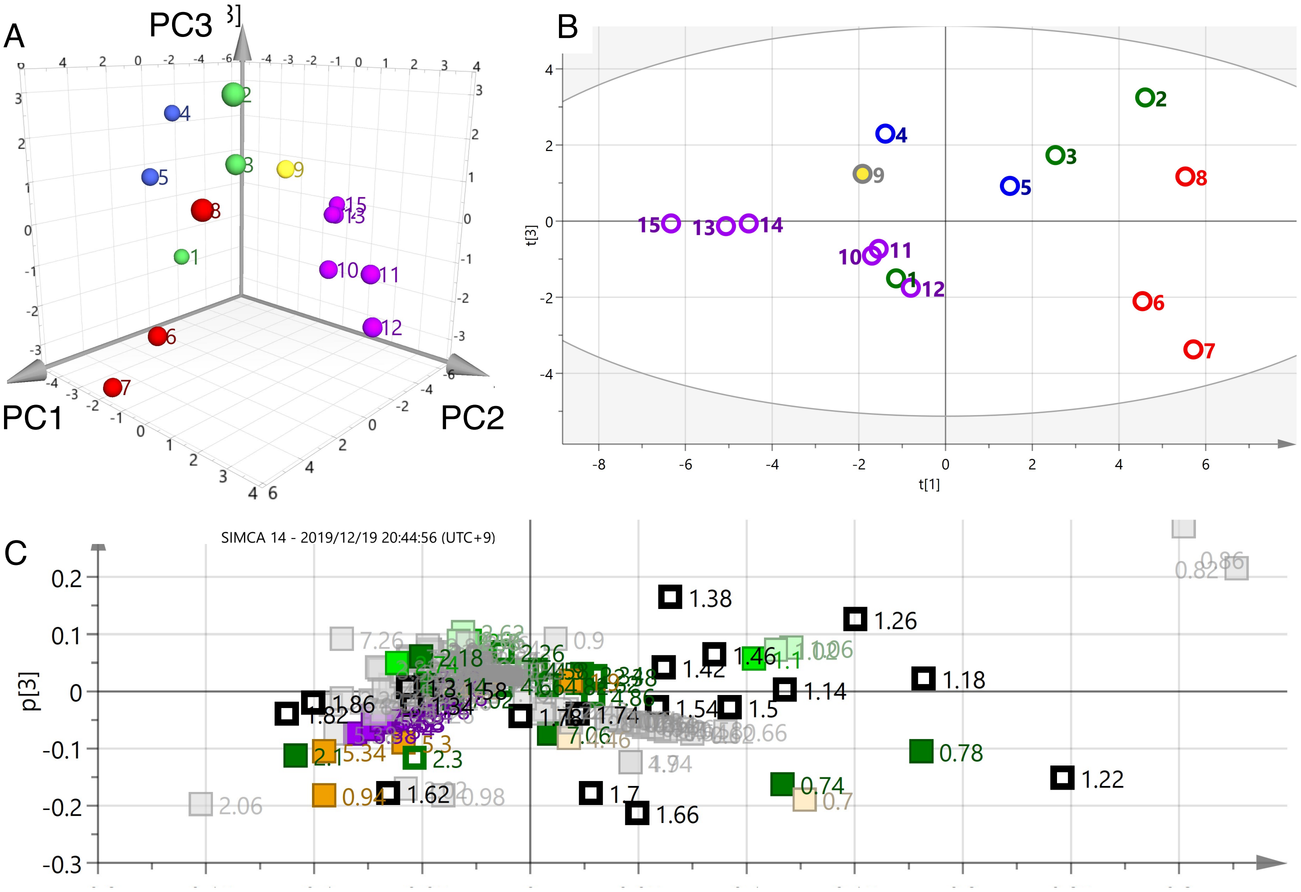

Figure S2. Principal component analyses (PCA) using ^{1}H-NMR spectrum extracted with $CDCl_3$ of different *Atratylodes* samples. (A) 3D Score plot of PC1, PC2 and PC3 scores, (B) Score plot of PC1 and PC3 scores, (C) Loading plot for PC1 and PC3 components.

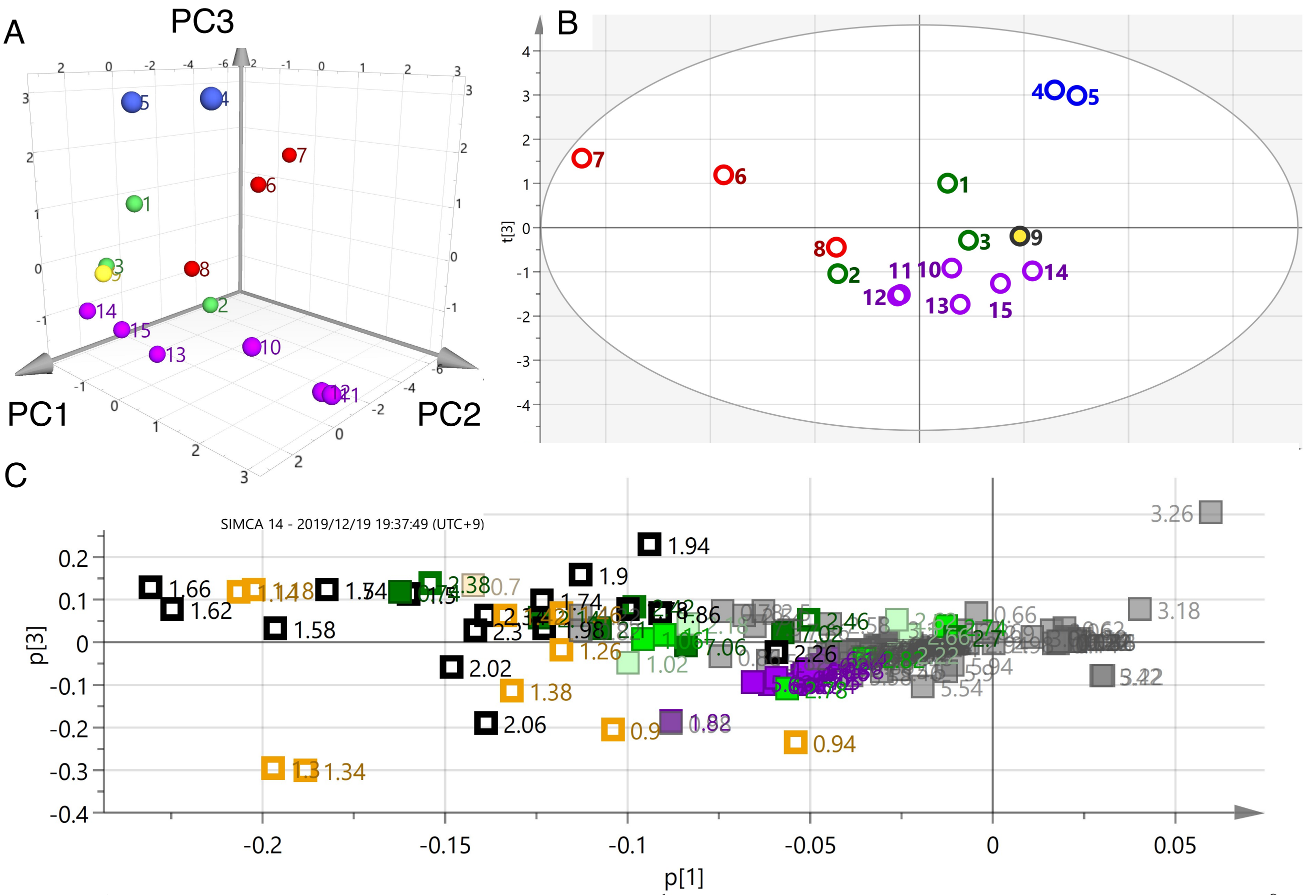

Figure S3. Principal component analyses (PCA) using ^{1}H-NMR spectrum removed a sugar-based region (δ 3.30–5.40) extracted with CD_3OD of different *Atratylodes* samples. (A) 3D Score plot of PC1, PC2 and PC3 scores, (B) Score plot of PC1 and PC3 scores, (C) Loading plot for PC1 and PC3 components.
